# Health of Cities

**Published:** 1857-09

**Authors:** 


### HEALTH OF CITIES.

| | Population. | Size in Acres. | Park Reserves. | To each House. | Mortality. |
|---|---|---|---|---|---|
| London, | 2¼ millions, | 76,000, | 1537, | seven, | 1 in 41. |
| New York, | ¾ million, | 14,000, | 950, | thirteen, | 1 in 34. |
| Philadelphia, | ½ million, | 70,000, | | six, | 1 in 50. |

London includes five times as much space as New York, with only one-third more park room. "CENTRAL PARK," containing seven hundred and seventy-six acres, is just double the size of *Hyde Park*, the boast of England. Leaving out Hyde Park, the Central Park is larger than any other two parks in London. The Croton water is the purest supplied to any large city in the world. Yet, with the purest airage and the finest water, more persons die every year in New York than in any other large city on the surface of the globe, whose statistics are known. What is the reason? It is found in our dwellings. Too many people live in the same house. Too many sleep in small, dismal, ill-ventilated rooms, cellars, attics, and the middle one *of three* rooms deep.

We wish that the Editors of the *Scalpel* and *Medical Gazette*, whose heads are larger and whose experience has run through more years than ours, would lend their minds to the practical inquiry, why is New York the highest of all large cities in its mortality, notwithstanding its superior advantages as to drainage, park room and sea air, to say nothing of the six miles of running water area on its eastern and western boundaries? It will not do to say that our city is filthier as to its streets than other cities, nor to attribute the increased mortality to the larger number of persons who land here from abroad, because the foreign mortality at all the public institutions for eighteen hundred and fifty-five was fourteen hundred and seventy, of which number thirteen hundred and five were from the Emigration Hospital at Ward's Island, where poor sick immigrants are sent on arriving here. That is, of the 136,000 immigrants during 1855, there were 1305 passenger deaths, out of the entire foreign death 6103, and an entire mortality of 23,042. This is a great practical question, and we wonder that it has not engaged the attention of medical pens to a greater extent than it has done. If, on examination, it is found that crowded houses, tenement dwellings, and confined dormitories, are the demonstrable causes, then let corresponding efforts be made to remedy the evil.

Night itself predisposes to disease, and sleep adds largely to that predisposition, by reason of its slow breathing and languid circulation; and if in that highly predisposed state, we, for a third of our existence, breathe a vitiated atmosphere, vitiated by that heavy dampness which characterizes the night time, to say nothing of the most disagreeable closeness which is perceptible on entering the sleeping apartment of even one person, in the morning, we cannot wonder that so many are sent to the grave every year, where this state of things prevails. The subject certainly commends itself to the mature reflection of the humane, for it is a higher humanity by far to prevent disease, than to build hospitals and found asylums. No one should sleep in a room smaller than twelve feet square, nor lower than the second floor, and even then, a fireplace, and a door or window, should be open, even in the country.

